# Experience with a second language affects the use of fundamental frequency in speech segmentation

**DOI:** 10.1371/journal.pone.0181709

**Published:** 2017-07-24

**Authors:** Annie Tremblay, Jui Namjoshi, Elsa Spinelli, Mirjam Broersma, Taehong Cho, Sahyang Kim, Maria Teresa Martínez-García, Katrina Connell

**Affiliations:** 1 Department of Linguistics, University of Kansas, Lawrence, Kansas, United States of America; 2 Department of French and Italian, University of Illinois, Urbana, Illinois, United States of America; 3 Laboratoire de Psychologie et Neurocognition, Université Grenoble Alpes, Grenoble, France; 4 Centre for Language Studies, Radboud University, Nijmegen, The Netherlands; 5 Department of English Language and Literature, Hanyang University, Seoul, Korea; 6 Department of English Education, Hongik University, Seoul, Korea; 7 Department of Occidental Languages, Hankuk University of Foreign Studies, Seoul, Korea; Universitat Zurich, SWITZERLAND

## Abstract

This study investigates whether listeners’ experience with a second language learned later in life affects their use of fundamental frequency (F0) as a cue to word boundaries in the segmentation of an artificial language (AL), particularly when the cues to word boundaries conflict between the first language (L1) and second language (L2). F0 signals phrase-final (and thus word-final) boundaries in French but word-initial boundaries in English. Participants were functionally monolingual French listeners, functionally monolingual English listeners, bilingual L1-English L2-French listeners, and bilingual L1-French L2-English listeners. They completed the AL-segmentation task with F0 signaling word-final boundaries or without prosodic cues to word boundaries (monolingual groups only). After listening to the AL, participants completed a forced-choice word-identification task in which the foils were either non-words or part-words. The results show that the monolingual French listeners, but not the monolingual English listeners, performed better in the presence of F0 cues than in the absence of such cues. Moreover, bilingual status modulated listeners’ use of F0 cues to word-final boundaries, with bilingual French listeners performing less accurately than monolingual French listeners on both word types but with bilingual English listeners performing more accurately than monolingual English listeners on non-words. These findings not only confirm that speech segmentation is modulated by the L1, but also newly demonstrate that listeners’ experience with the L2 (French or English) affects their use of F0 cues in speech segmentation. This suggests that listeners’ use of prosodic cues to word boundaries is adaptive and non-selective, and can change as a function of language experience.

## Introduction

Research has shown that upon hearing an unfamiliar language, listeners use all the cues that are reliable predictors of word boundaries in their native language (L1) to segment the unfamiliar language into individual words [1–11]. What is unclear from this research, however, is whether (and if so, *how*) the learning of a second language (L2) later in life impacts listeners’ use of cues for segmenting a new or unfamiliar language: Do bilingual listeners rely strictly on segmentation cues from their L1? Alternatively, if late-acquired L2 experience affects the segmentation of a new language, in cases where the same cue signals different word boundaries in the L1 and the L2 (e.g., word-final boundaries in the L1 and word-initial boundaries in the L2), would bilingual listeners select the strategy that turns out to be the most successful (from either the L1 or the L2) for segmenting the new language, or would they show some compromise in the degree with which they can use either strategy (since they conflict across the two languages)?

Investigating whether the late learning of an L2 impacts the segmentation of a new language would elucidate not only whether listeners’ use of segmentation strategies is *adaptive* (i.e., it can change as a function of experience with an L2), but also whether it is *selective* (i.e., listeners can select which of the L1 or L2 segmentation strategy to use based on its success for segmenting the new language). Importantly, understanding *how* the learning of an L2 influences the segmentation of an unfamiliar language will have significant implications for research on the early acquisition of a third language.

The present study seeks to answer these questions by investigating whether listeners’ experience with an L2 learned later in life affects their use of fundamental frequency (F0) as a cue to word boundaries in the segmentation of an artificial language (AL). In typical AL segmentation paradigms conducted with adult listeners, participants listen to a continuous string of syllables sequenced such that some syllables always co-occur (and thus form a word) and others only occasionally or never co-occur (and thus do not form a word). During exposure to such ALs, adult listeners extract transitional probabilities between syllables (via statistical learning mechanisms) and tend to perform above chance when deciding which of two auditory strings was a word in the AL [12–14]. Importantly, when prosodic cues that are informative to identify word boundaries in the L1 also occur in the AL, listeners’ performance is better than without such cues [1, 4–8, 10, 11].

This study examines listeners’ use of F0 in the segmentation of an AL as a function of their L1 and L2 experience, particularly when the cues to word boundaries conflict between the L1 and the L2. The two languages that are the focus of the present study are French and English, which differ in how F0 signals word boundaries. Although French does not have lexical stress, it has phrasal prosody, with phrases in sentence-internal position ending with an F0 rise and thus with words in phrase-final position having their final boundaries signaled by an F0 rise [15, 16]. French listeners have indeed been shown to use this F0 rise to locate *word-final* boundaries in continuous French speech [17–20] and in ALs [1, 10]. By contrast, in English, an F0 rise generally signals stressed syllables in accented words [21], and most English words are stressed word-initially [22, 23]. Thus, an F0 rise tends to signal *word-initial* boundaries in English, and English listeners use this F0 rise to locate word-initial boundaries in an AL [10].

The present study compares listeners’ performance in the use of F0 cues to word-final boundaries in an AL as a function of both their L1 and L2 experiences. More specifically, it examines the use of F0 cues to word-final boundaries in native French listeners with little knowledge of English (henceforth referred to as “functionally monolingual French listeners”), native English listeners with little knowledge of French (henceforth referred to as “functionally monolingual English listeners”), native French listeners who learned English later on in life and are at a relatively high proficiency in English (henceforth referred to as “L1-French L2-English listeners”), and native English listeners who learned French later on in life and are at a relatively high proficiency in French (henceforth referred to as “L1-English L2-French listeners”). In doing so, the present study newly tests: (i) whether L1-English listeners with experience in L2-French can use F0 cues to locate word-final boundaries in the AL (unlike functionally monolingual English listeners); and (ii) whether L1-French listeners with experience in L2-English show a decline in their ability to use F0 cues to word-final boundaries in the AL (as compared to functionally monolingual French listeners).

Finding that L1-English L2-French listeners make greater use of F0 cues to word-final boundaries in the AL than monolingual English listeners would suggest that listeners’ use of segmentation strategies is *adaptive*, and thus can change as a result of L2 experience. Moreover, finding that L1-French L2-English listeners are less successful than monolingual French listeners at using F0 cues to word-final boundaries would suggest that listeners’ use of segmentation strategies is *non-selective*: When listeners have knowledge of two segmentation strategies that conflict in how they track word boundaries in different languages (e.g., F0 rise as a cue to word-initial boundaries in English but word-final boundaries in French), they do not select which segmentation strategy to adopt as a function of how useful this strategy would be to segment the AL; instead, they are less accurate in using either strategy (as opposed to selecting the segmentation strategy that would work for segmenting the AL, here the French strategy). Such findings would suggest that bilingual listeners’ segmentation of an unfamiliar language is influenced by their experience with both the L1 and the L2, and that strategies cannot be selected as a function of how useful they are for segmenting the unfamiliar language. Additionally, if the amount of L2 experience modulates bilingual listeners’ segmentation strategies, we may expect to find a relationship between bilingual listeners’ use of F0 cues to word-final boundaries in the AL and the extent of their experience with L2-French or L2-English.

The present study is a replication of Kim, Broersma and Cho (4) (a study on Dutch and Korean listeners’ use of prosodic cues in AL segmentation) but with French and English listeners, using two of the conditions from Kim, Broersma and Cho (4), namely an AL with F0 cues to word-final boundaries and one with no prosodic cues to word boundaries. As in Kim, Broersma and Cho (4), in the forced-choice word-identification test following the AL listening phase, this study used both non-words and part-words as foils. Non-words are syllables that were present in the AL but that were never heard consecutively; part-words are syllables that were heard consecutively in the AL, with the first or last two syllables belonging to a word and the remaining syllable belonging to an adjacent word in the AL. Saffran, Aslin and Newport (13) found that adult listeners were more accurate in identifying words when the foil was a non-word than when the foil was a part-word, but they were above chance on both word types. Performance on words accompanied by non-word foils was interpreted as reflecting listeners’ ability to extract the probabilities of co-occurrence of two syllables, whereas performance on words accompanied by part-word foils was interpreted as reflecting listeners’ ability to extract the conditional probabilities of successive syllables (e.g., the probability that if two syllables co-occur, then they should be followed by a given third syllable) (for discussion, see [24, 25]). The present study includes both types of foils in order to assess how listeners’ experience with an L2 modulates their ability to extract both the probabilities of co-occurrence of two syllables and the conditional probabilities of successive syllables when segmenting an AL into units.

## Method

The research protocol followed in this study and the written consent obtained from all participants was approved by the University of Kansas Human Subjects Committee (IRB ID: 20493).

### Participants

A total of 68 native French listeners and 61 native English listeners participated in this study. All French listeners and all English listeners had parents who spoke, respectively, only French or only English as native language, and none were exposed to languages other than their native language (i.e., respectively, French or English) prior to the age of 12.

Of the French listeners, 48 were functionally monolingual: They lived in France at the time of testing and reported having limited or no knowledge of English. The remaining 20 French listeners were L1-French L2-English bilinguals: They lived in the US at the time of testing and had high proficiency in English, and they reported having spent on average 17 months (range: 3–75) in the US since their last stay of at least 3 months in France.

All English listeners were in the US at the time of testing. Of them, 40 were “functionally monolingual”: They reported having limited or no knowledge of French (and no knowledge of Korean, which patterns similarly to French with respect to F0; for discussion, see [20]). The remaining 21 English listeners were L1-English L2-French bilinguals: They had learned French as L2 and reached high proficiency in French (performance on a cloze test: 33/45, which is considered advanced; [26]).

This study used a between-subjects design, with each participant being exposed either to the AL without any prosodic cues to word boundaries or to the AL with F0 cues to word-final boundaries. The distribution of participants across conditions was as follows: 23 monolingual French listeners heard the AL with no prosodic cues, and 25 the AL with F0 cues to word-final boundaries; all L1-French L2-English listeners heard the AL with F0 cues to word-final boundaries; 20 monolingual English listeners heard the AL with no prosodic cues, and 20 the AL with F0 cues to word-final boundaries; all L1-English L2-French listeners heard the AL with F0 cues to word-final boundaries. None of the bilinguals were assigned to an AL with no prosodic cues to word-final boundaries, because there was no reason to assume that monolingual and bilingual listeners would differ in their use of transitional probabilities alone. Table 1 presents the participants’ age (in years), sex distribution (number of females), and self-reported weekly use of French (participants specified what percentage of the time they used French in a week) separately for each group.

**Table 1:** Participants’ biographical information and experimental condition.

AL with only transitional probabilities				AL with F0 cues to word-final boundaries			
Group	Age (yrs^a^)	Number of Females	Weekly Use of French (%^a^)	Group	Age (yrs^a^)	Number of Females	Weekly Use of French (%^a^)
1. Functionally Monolingual French Listeners (*n* = 23)	19.1 (1.3)	21	96.4% (6.3%)	3. Functionally Monolingual French Listeners (*n* = 25)	21.5 (4.5)	23	91.7% (10.5%)
2. Functionally Monolingual English Listeners (*n* = 20)	22.8 (3.3)	13	n/a	4. Functionally Monolingual English Listeners (*n* = 20)	25.6 (5.2)	9	n/a
				5. L1-French L2-English Bilingual Listeners (*n* = 20)	25.3 (4.3)	13	37.5% (17.1%)
				6. L1-English L2-French Bilingual Listeners (*n* = 21)	26.9 (5.6)	13	19.1% (14.6%)

^a^ Mean (standard deviation)

### Materials

**Fig 1:**
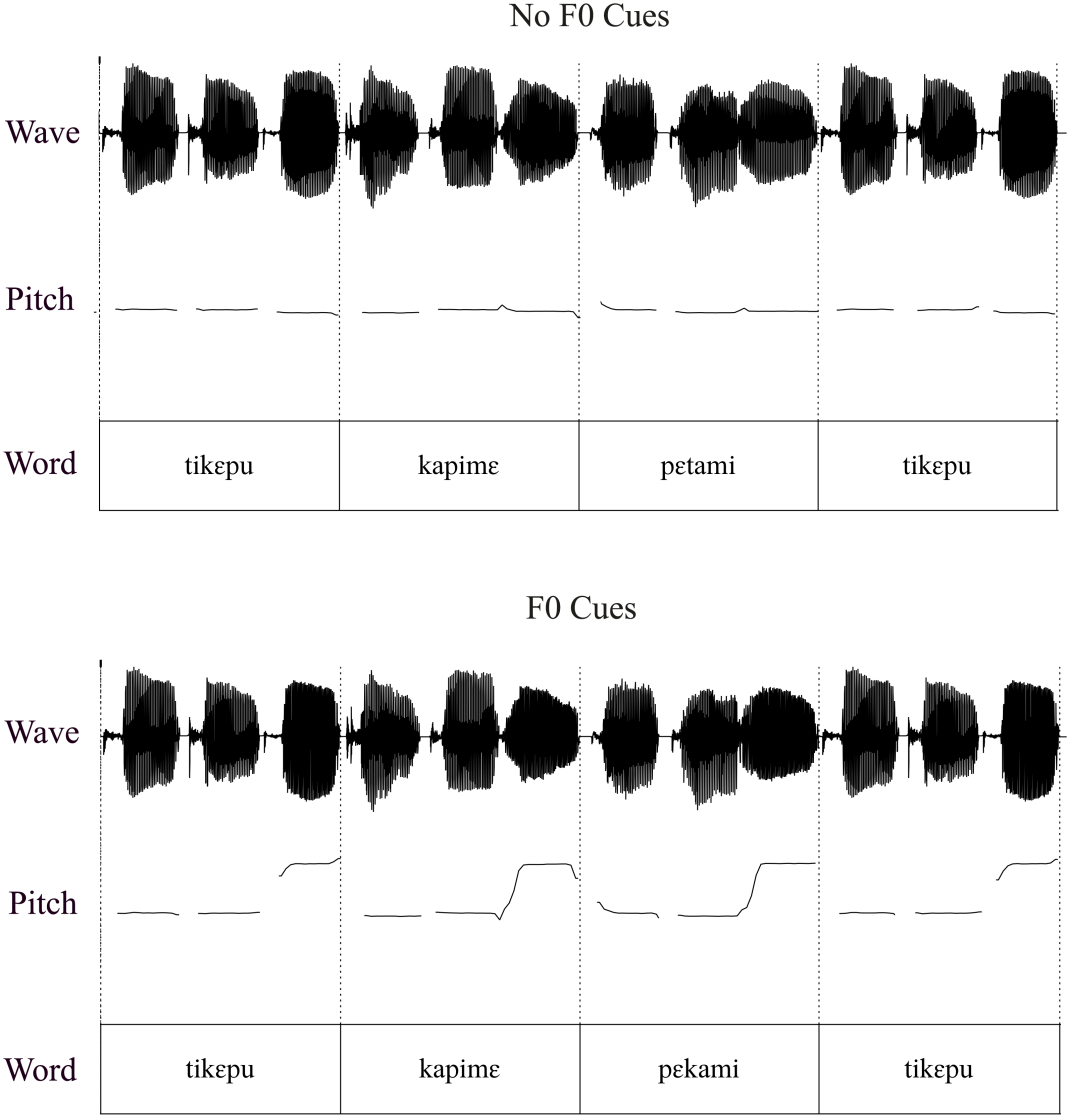
Schematic illustration of words in the AL without F0 cues (top panel) and the AL with F0 cues (bottom panel). In each panel, the top row represents the sound wave, the middle row the pitch track, and the bottom row the words.

This study used the same stimuli and audiorecordings as those used in Kim, Broersma and Cho (4). In the exposure phase of the experiment, participants listened to an AL speech stream that contained six trisyllabic words: [tikεpu], [pεtami], [mupaki], [kapimε], [kutεpa], [pimatu]. None of the trisyllabic words were French or English words. The syllables were recorded in isolation by a female native speaker of Korean. All syllables had their duration neutralized to 252 ms. The syllables were then combined to create the six trisyllabic words. In the AL with only transitional probabilities (i.e., without prosodic cues to word boundaries), the words had a flat F0 of 190 Hz; in the AL where F0 signaled word-final boundaries, for each word, the first two syllables had a flat F0 of 190 Hz and the last syllable had a flat F0 of 250 Hz. A schematic illustration of the words in the ALs without and with F0 cues is provided in Fig 1. Each word was heard 126 times throughout the AL. No single word occurred consecutively, and there was no pause between the words. Syllable-to-syllable transitional probabilities ranged from 0.5 to 1 within words and from 0.03 to 0.44 between words. There were 20-second intensity fade-in and fade-out periods at the beginning and end of the stream. Additional details about the AL can be found in Kim, Broersma and Cho (4).

In the test phase of the experiment, the participants heard 36 pairs of trisyllabic sequences. These 36 pairs were created by comparing the six AL words to six foils consisting of trisyllabic sequences that did not form a word: Three foils were non-words (i.e., containing three syllables that were present in the AL but none of which were heard consecutively in the AL), and three foils were part-words (i.e., containing two syllables that were heard consecutively in the AL, with the first or last two syllables belonging to a word and the remaining syllable belonging to an adjacent word in the AL). All syllables in the test phase had an F0 of 190 Hz. Additional details about the stimuli used in the test phase of the experiment can be found in Kim, Broersma and Cho (4).

The voiceless stops produced by the native Korean speaker in Kim, Broersma and Cho (4) and used in the present study had a mean Voice Onset Time (VOT) of 39 ms. Although this VOT is closer to prototypical English voiceless stops than to prototypical French voiceless stops, this does not pose a concern for the present study for the following reasons: First, even for French listeners, the stops could only be assimilated to voiceless stops (albeit bad exemplars of the French voiceless stops); second, neither the familiarization phase nor the testing phase included voiced stops—hence, performance was not contingent on voiceless stops being distinguished from voiced stops; third, as will be shown in the results, the monolingual English listeners did not outperform the monolingual French listeners in the control condition (with a flat F0), suggesting that the more English-like VOT in the stimuli did not enhance English listeners’ performance or adversely affect French listeners’ performance. Thus, the use of a more English-like VOT does not undermine the design of this study or its results.

### Procedures

Participants completed the experiment in a quiet space in a laboratory. In the exposure phase of the experiment, they listened to the AL twice. The total duration of the exposure phase for each participant was exactly 19 minutes and 4 seconds. The exposure phase was immediately followed by the testing phase. In the testing phase, participants heard pairs of word (with no prosodic cues to word boundaries) separated by an interstimulus interval of 800 ms, and identified which of the two words they thought they had heard in the AL by selecting 1 or 2 (corresponding to the first and second words). Their accuracy in selecting the correct word was recorded. The complete experiment took approximately 25 minutes.

### Data analysis

Participants’ accuracy was analyzed with logit mixed-effects models using the lme4 package of R [27]. For each participant group, we ran logit mixed-effects models comparing listeners’ accuracy on each word type to chance. Using separate logit mixed-effects models, we also examined the effects of F0 (no F0 cues, F0 cues), foil type (non-word, part-word), L1 (French, English), and two- and three-way interactions on participants’ accuracy. We first analyzed the accuracy of all monolingual groups (Groups 1–4 in Table 1). The initial model included F0 (no F0 cues, F0 cues), foil type (non-word, part-word), L1 (French, English), and their interaction as fixed effects, and participant and item as random intercepts (models with random slopes did not converge). The factors in this analysis were dummy coded, and in the presence of significant interactions, simple effects were examined by releveling the model. Next, we analyzed the accuracy of all groups who heard the AL with F0 cues to word-final boundaries (Groups 3–6 in Table 1). The initial model included L1 (French, English), foil type (non-word, part-word), bilingual status (no, yes), and their interaction as fixed effects, and participant and item as random intercepts. The factors in this analysis were also dummy coded, and in the presence of significant interactions, simple effects were examined by releveling the model. For both analyses, fixed effects were removed from the model one at a time, and model comparisons were run in pairwise fashion to determine if the more complex model accounted for significantly more of the variance (α = .05), as determined by log-likelihood ratio tests. Using this backward-fitting method, we report the model that accounted for significantly more of the variance than simpler models. Finally, to ascertain whether the observed effects of bilingual status can indeed be attributed to listeners’ experience with the L2, using logit mixed-effects models with the same random effects, we examined the relationship between the L1-French L2-English listeners’ accuracy and their length of stay in the US (in months), and between the L1-English L2-French listeners’ accuracy and their proficiency in French (cloze test scores).

## Results

The raw data upon which the following analyses are based can be found in the S1 File (Data.csv).

### Functionally monolingual listeners: AL with vs. without F0 cues

**Fig 2:**
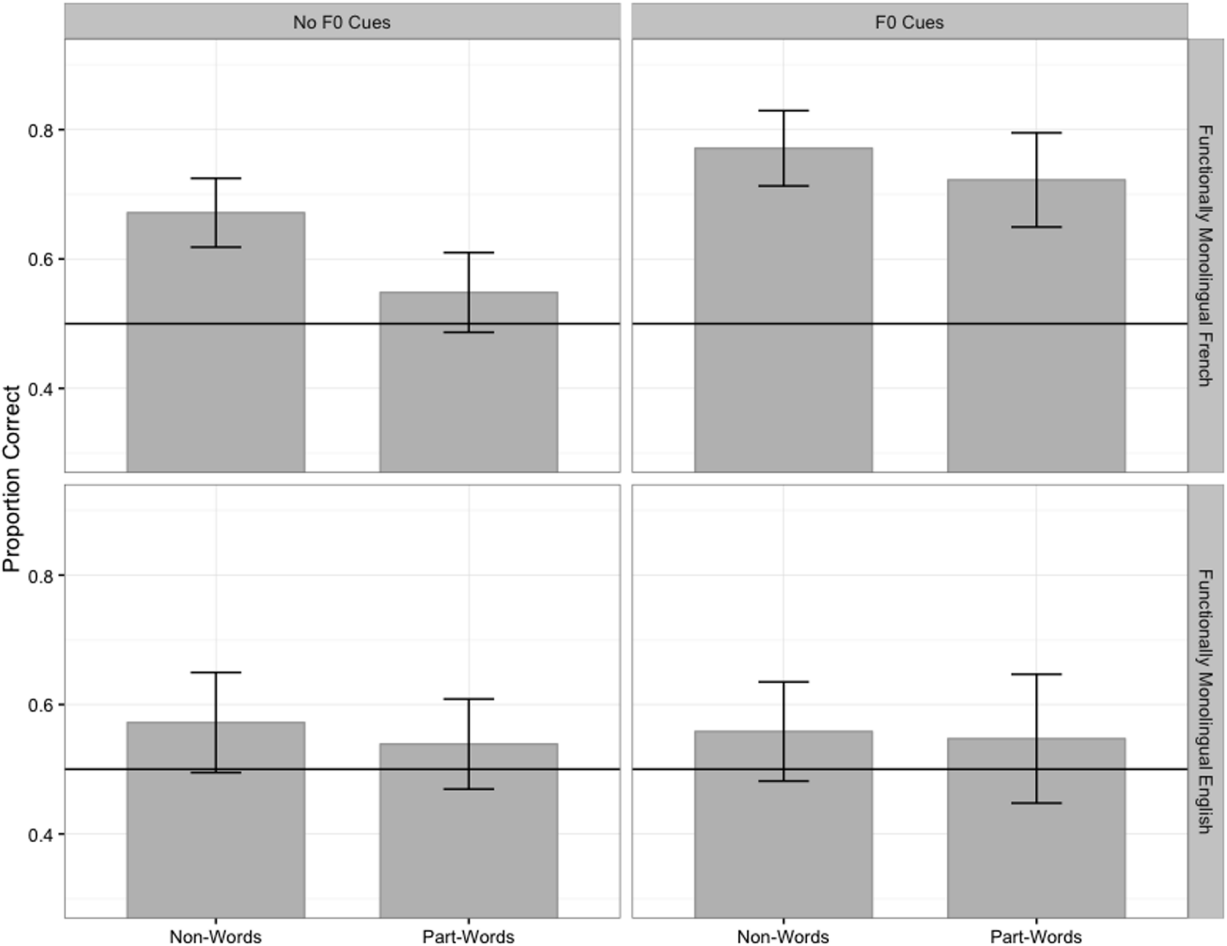
Functionally monolingual French and English listeners’ mean proportions of correct responses in the conditions without and with F0 cues to word-final boundaries. The error bars represent one standard error of the mean; the horizontal line represents chance performance.

First, to determine whether the L1 modulates listeners’ use of F0 cues to word boundaries, we examine whether the functionally monolingual French and English listeners differed in their performance when the AL contained or did not contain F0 cues to word-final boundaries. Fig 2 presents the monolingual listeners’ mean proportions of correct responses on non-word vs. part-word foils in the AL conditions with vs. without F0 cues to word-final boundaries (Groups 1–4 in Table 1).

For the non-word foils, logit mixed-effects models comparing the monolingual listeners’ accuracy to chance revealed that the monolingual French listeners performed significantly above chance both when the AL did not contain F0 cues to word-final boundaries (*β* = 0.77, *z* = 3.8, *p* < .001) and when it contained such cues (*β* = 1.31, *z* = 6.4, *p* < .001); conversely, the monolingual English listeners did not perform significantly differently from chance when the AL did not contain F0 cues to word-final boundaries (*β* = 0.32, *z* = 1.52, *p* > .1) or when it contained such cues (*β* = 0.25, *z* = 1.22, *p* > .1). For the part-word foils, similar logit mixed-effect models comparing the monolingual listeners’ accuracy to chance revealed that only the monolingual French listeners who heard the AL with F0 cues to word-final boundaries performed significantly above chance (*β* = 1.05, *z* = 6, *p* <. 001); none of the remaining groups performed significantly differently from chance (monolingual French listeners, no F0 cues: *β* = 0.21, *z* = 1.22, *p >* .1; monolingual English listeners, no F0 cues: *β* = 0.17, *z* <|1|, *p* > .1; monolingual English listeners, F0 cues: *β* = 0.22, *z* = 1.18, *p* > .1).

Logit mixed-effects models were conducted on the functionally monolingual listeners’ accuracy to examine the effects of F0 (no F0 cues, F0 cues), foil type (non-word, part-word), L1 (French, English), and their interaction. The baseline was French listeners’ performance on non-word foils in the AL with no F0 cues. The model with the best fit included the simple effects of F0, foil type, and L1, as well as the interaction between F0 and L1. The estimate, standard error, *z* value, and *p* value associated with the fixed effects are presented in Table 2.

**Table 2:** Best logit mixed-effects model on accuracy of functionally monolingual listeners (French listeners’ performance on non-word foils in the AL with No F0 cues as baseline)^a^.

Effect	*β* (SE)	*z*	*p*
(Intercept)	0.60 (0.17)	3.74	< .001
F0	0.70 (0.18)	3.83	< .001
Foil Type	–0.26 (0.08)	–3.42	< .001
L1	–0.24 (0.19)	–1.27	> .1
F0 × L1	–0.71 (0.27)	–2.64	.008

^a^ *df* = 3164, 88 participants, 6 items.

The model results summarized in Table 2 indicate that the monolingual French listeners’ performance on non-word foils in the condition with no F0 cues was larger than 0 (intercept); their performance on non-word foils was higher in the condition with F0 cues than in the condition with no F0 cues (simple effect of experimental condition); and their performance in the condition with no F0 cues was lower on part-word foils than on non-word foils (simple effect of foil type). The lack of interaction between foil type and F0 indicates that the simple effect of F0 is true of both foil types (and the simple effect of foil type is true for both F0 conditions), and the lack of interaction between foil type and L1 suggests that the simple effect of L1 is true of both foil types (and the simple effect of foil type is true for both L1s). The model also yielded a significant two-way interaction between F0 and L1.

To understand the nature of the significant two-way interaction, the model was releveled such that *English* listeners’ performance on non-word foils in the AL with no F0 cues would be the baseline. This baseline selection allowed us to examine the simple effect of F0 for English listeners rather than for French listeners (with non-word foils), and thus made it possible to determine whether the present experimental design was appropriate for use with French and English listeners (if so, an effect of F0 should be found for French listeners but not for English listeners). The estimate, standard error, *z* value, and *p* value associated with the fixed effects in this releveled model are presented in Table 3.

**Table 3:** Best logit mixed-effects model on accuracy of functionally monolingual listeners (English listeners’ performance on non-word foils in the AL with No F0 cues as baseline)^a^.

Effect	*β* (SE)	*z*	*p*
(Intercept)	0.37 (0.17)	2.18	.030
**F0**	**–0.004 (0.19)**	**< |1|**	**>. 1**
Foil Type	0.26 (0.08)	3.42	< .001
L1	0.24 (0.19)	1.27	> .1
F0 × L1	0.71 (0.27)	2.64	.008

^a^ *df* = 3164, 88 participants, 6 items;

the effect that differs (in significance) from those reported in Table 2 is presented in bold.

Of the results of the releveled model in Table 3, only one effect (in bold) differs (in significance) from the effects reported in Table 2: English listeners’ performance on non-word foils in the condition with no F0 cues did not differ from their performance on the same foils in the condition with F0 cues. Hence, the two-way interaction between F0 and L1 (in Tables 2 and 3) stemmed from the effect of F0 present in the French listeners’ data but absent from the English listeners’ data.

In summary, the functionally monolingual groups showed higher accuracy on non-word foils than on part-word foils, with performance on non-word foils being above chance for all groups when the AL did not contain F0 cues but only for the functionally monolingual French listeners when the AL contained F0 cues, and with performance on part-word foils being above chance only when functionally monolingual French listeners were exposed to the AL with F0 cues. Importantly, only the monolingual French listeners showed an effect of F0, performing better in the presence of F0 cues than in the absence of such cues, thus validating the use of this experimental design with French and English listeners.

### AL with F0 cues: Functionally monolingual vs. bilingual listeners

**Fig 3:**
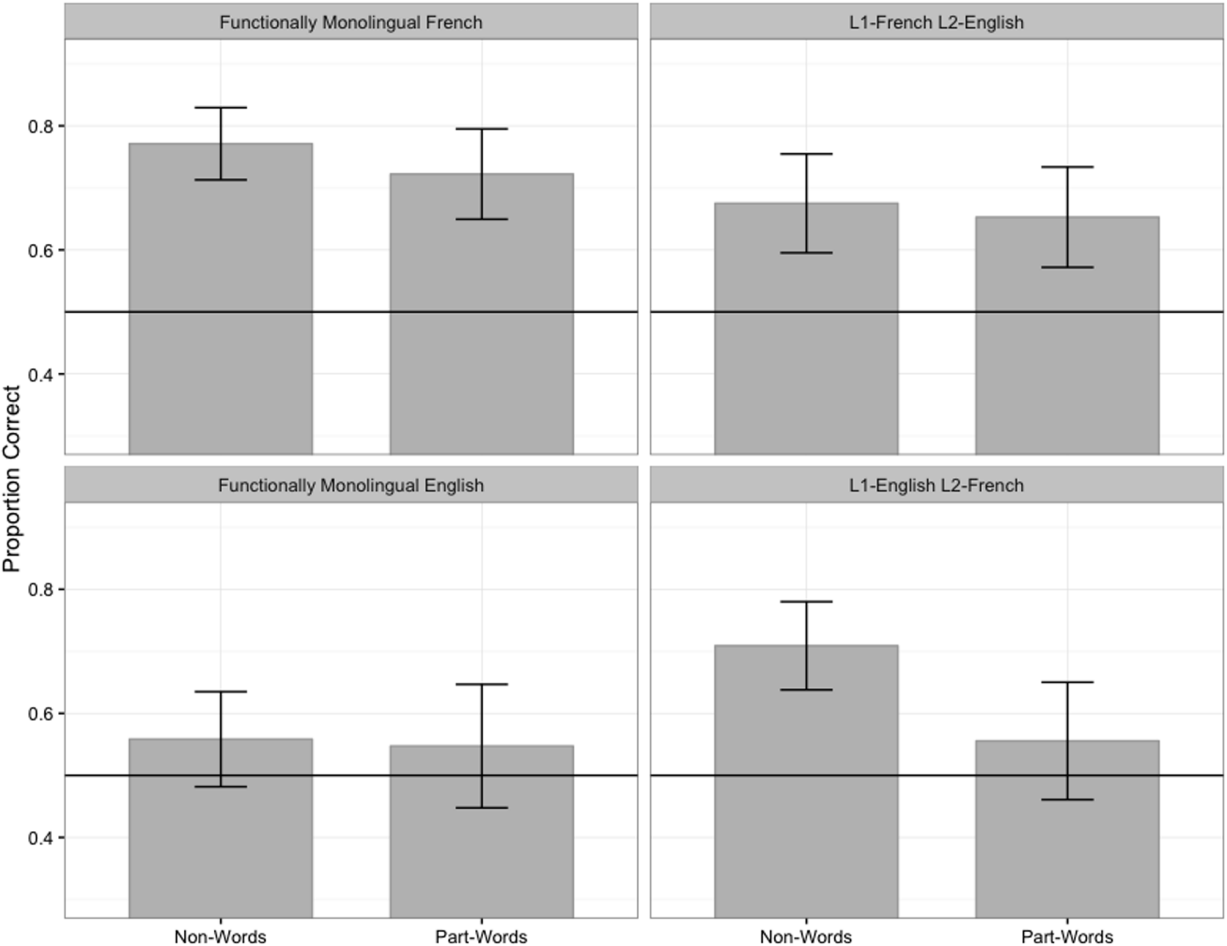
All listeners’ mean proportions of correct responses in the condition with F0 cues to word-final boundaries. The error bars represent one standard error of the mean; the horizontal line represents chance performance.

Next, to determine whether listeners’ experience with the L2 affects their use of F0 cues to word-final boundaries in the AL, we compare the performances of functionally monolingual listeners and bilingual listeners when the AL contained F0 cues to word-final boundaries. Fig 3 presents monolingual and bilingual listeners’ mean proportions of correct responses on non-word vs. part-word foils in the AL condition with F0 cues to word-final boundaries (Groups 3–6 in Table 1); the results of the monolingual groups with F0 cues (right panels of Fig 2) are repeated (left panels of Fig 3) for the sake of comparisons with the bilingual groups.

For non-word foils, logit mixed-effects models comparing the bilingual listeners’ accuracy to chance revealed that both bilingual groups performed significantly above chance (L1-French L2-English listeners: *β* = 0.8, *z* = 3.76, *p* < .001; L1-English L2-French listeners: *β* = 0.97, *z* = 4.6, *p* < .001). However, for part-word foils, only the L1-French L2-English group performed significantly above chance (*β* = 0.69, *z* = 3.64, *p* < .001); the L1-English L2-French group did not perform significantly differently from chance (*β* = 0.25, *z* = 1.4, *p >* .1).

Again, logit mixed-effects models were conducted on listeners’ accuracy in the condition with F0 cues to examine the effects of foil type (non-word, part-word), L1 (French, English), and bilingual status (monolingual, bilingual), and their interaction. The baseline was monolingual French listeners’ performance on non-word foils. The model with the best fit included all fixed effects. The estimate, standard error, *z* value, and *p* value associated with the fixed effects are presented in Table 4.

**Table 4:** Logit mixed-effects model with best fit on listeners’ accuracy in condition with F0 cues (with monolingual French listeners’ performance on non-word foils as baseline)^a^.

Effect	*β* (SE)	*z*	*p*
(Intercept)	1.35 (0.20)	6.73	< .001
Foil Type	–0.28 (0.16)	–1.77	.076
L1	–1.09 (0.26)	–4.19	< .001
Bilingual Status	–0.55 (0.26)	–2.09	.037
Foil Type × L1	0.23 (0.22)	1.04	> .1
Foil Type × Bilingual Status	0.18 (0.23)	<|1|	> .1
L1 × Bilingual Status	1.29 (0.37)	3.45	< .001
Foil Type × L1 × Bilingual Status	–0.86 (0.32)	–2.69	.007

^a^ *df* = 3096, 86 participants, 6 items.

The model results summarized in Table 4 indicate that monolingual French listeners’ performance on non-word foils was higher than 0 (intercept); monolingual French listeners’ performance showed a trend towards being lower on part-word foils than on non-word foils (marginal simple effect of foil type); monolingual English listeners’ performance on non-word foils was lower than monolingual French listeners’ performance on the same foils (simple effect of L1); and bilingual French listeners’ performance on non-word foils was lower than monolingual French listeners’ performance on the same foils (simple effect of bilingual status). The lack of interaction between foil type and L1 indicates that the simple effect of L1 was true of both foil types, and the lack of interaction between foil type and bilingual status suggests that the effect of bilingual status was true of both foil types. Importantly, the model also yielded a significant two-way interaction between L1 and bilingual status, and a significant three-way interaction between foil type, L1, and bilingual status.

To understand the nature of the significant two- and three-way interactions, the model was releveled such that monolingual *English* listeners’ performance on non-word foils would be the baseline. This baseline selection allowed us to examine the simple effect of bilingualism for English listeners rather than for French listeners (with non-word foils), and thus made it possible to determine whether experience with an L2 affects the segmentation of an AL for both L1-French and L1-English listeners (if so, an effect of bilingualism should be found for both groups). The estimate, standard error, *z* value, and *p* value associated with the fixed effects in this releveled model are presented in Table 5.

**Table 5:** Logit mixed-effects model with best fit on listeners’ accuracy in condition with F0 cues (with monolingual English listeners’ performance on non-word foils as baseline)^a^.

Effect	*β* (SE)	*z*	*p*
**(Intercept)**	**0.26 (0.21)**	**1.25**	**> .1**
**Foil Type**	**–0.05 (0.16)**	**< |1|**	**> .1**
L1	1.09 (0.26)	4.19	< .001
**Bilingual Status**	**0.74 (0.28)**	**2.79**	**.005**
Foil Type × L1	–0.23 (0.22)	–1.04	> .1
**Foil Type × Bilingual Status**	**–0.69 (0.23)**	**–3.05**	**.002**
L1 × Bilingual Status	–1.29 (0.37)	–3.45	< .001
Foil Type × L1 × Bilingual Status	0.86 (0.32)	2.69	.007

^a^ *df* = 3096, 86 participants, 6 items;

the effects that differ (in significance or directionality) from those reported in Table 4 are presented in bold.

Of the results of the releveled model in Table 5, four effects (in bold) differ (in significance or directionality) from those reported in Table 4: monolingual English listeners’ performance on non-word foils was not significantly different from 0 (intercept); monolingual English listeners’ performance on non-word foils did not differ from their performance on part-word foils (no simple effect of foil type); L1-English L2-French listeners’ performance on non-word foils was *higher* than monolingual English listeners’ performance on the same foils (simple effect of bilingual status); and the effect of bilingual status differed for the two foil types (interaction between foil type and bilingual status). Thus, the two-way interaction between bilingual status and L1 (in Tables 4 and 5) stemmed from the different directionality of the effect of bilingualism in the French and English listeners’ data, and the three-way interaction between foil type, L1, and bilingual status (in Tables 4 and 5) stemmed from the two-way interaction between foil type and bilingual status present in the L1-English listeners’ data but absent from the L1-French listeners’ data.

To understand the nature of the two-way interaction between foil type and bilingual status in English listeners’ data, the model was releveled once more such that monolingual English listeners’ performance on *part-word* foils would be the baseline. This baseline selection allowed us to examine the simple effect of bilingualism in English listeners’ data for part-word foils rather than for non-word foils, thus shedding light on whether bilingualism differentially affects English listeners’ performance on both foil types. The estimate, standard error, *z* value, and *p* value associated with the fixed effects in this releveled model are presented in Table 6.

**Table 6:** Logit mixed-effects model with best fit on listeners’ accuracy in condition with F0 cues (with monolingual English listeners’ performance on part-word foils as baseline)^a^.

Effect	*β* (SE)	*z*	*p*
(Intercept)	0.21 (0.21)	1.024	> .1
Foil Type	0.05 (0.16)	< |1|	> .1
L1	0.86 (0.26)	3.33	< .001
**Bilingual Status**	**0.06 (0.27)**	**< |1|**	**> .1**
Foil Type × L1	–0.23 (0.22)	–1.04	> .1
Foil Type × Bilingual Status	–0.69 (0.23)	–3.05	.002
L1 × Bilingual Status	–1.29 (0.37)	–3.45	< .001
Foil Type × L1 × Bilingual Status	0.86 (0.32)	2.69	.007

^a^ *df* = 3096, 86 participants, 6 items;

the effect that differs (in significance) from those reported in Table 5 is presented in bold.

Of the results of the releveled model in Table 6, only one effect (in bold) differs (in significance) from the effects reported in Table 5: Bilingual English listeners’ performance on part-word foils did not differ from monolingual English listeners’ performance on the same foils (no simple effect of bilingual status). Hence, the two-way interaction between foil type and bilingual status in L1-English listeners’ data (Table 5) stemmed from the effect of bilingualism on non-word foils but not on part-word foils.

To summarize, in the presence of F0 cues in the AL, both L1-French L2-English and L1-English L2-French listeners performed above chance on non-word foils, but only the L1-French L2-English group also performed above chance on part-word foils. Crucially, whereas L1-French L2-English listeners performed less accurately than monolingual French listeners on both foil types, L1-English L2-French listeners performed more accurately than monolingual English listeners only on non-word foils.

### AL with F0 cues: Experiential predictors of bilingual listeners’ performance

Finally, and importantly, to ascertain whether the observed effects of bilingual status can indeed be attributed to listeners’ experience with the L2, using similar logit mixed-effects models, we examined the relationship between the L1-French L2-English listeners’ accuracy and their length of stay in the US (in months), and between the L1-English L2-French listeners’ accuracy and their proficiency in French (cloze test scores). Given the three-way interaction between foil type, L1, and bilingual status observed in the previous analyses, these models also included foil type and the interaction between foil type and length of stay in the US or proficiency in French as fixed effects. In these analyses, the continuous variables were centered to reduce collinearity effects in the models and so that the effect of foil type would be interpreted as a main effect rather than as a simple effect. Three L1-French L2-English listeners were excluded from this analysis because of missing information about their length of stay in the US.

For the L1-French L2-English listeners, the model with the best fit included the effect of foil type and listeners’ length of stay in the US, but not their interaction; only the effect of length of stay in the US reached significance (*β* = –0.02, *z* = –2.55, *p* < .011), with participants showing lower accuracy in the condition with F0 cues as their stay in the US increased (effect of foil type: *β* = –0.15, *z* < |1|, *p* > .1). The lack of interaction between foil type and length of stay in the US indicates that L1-French L2-English listeners’ accuracy *on both foil types* decreased as length of time in the US increased. For the L1-English L2-French listeners, the best model included both the effect of foil type and the effect of proficiency, but not their interaction; the model yielded a main effect of foil type (*β* = –0.81, *z* = –4.85, *p* < .001), with participants showing lower accuracy on part-word foils than on non-word foils, and a simple effect of proficiency (*β* = 0.06, *z* = 1.97, *p* < .049), with participants being more accurate with increasing proficiency in French. Again, the lack of interaction between foil type and proficiency indicates that L1-English L2-French listeners’ accuracy *on both foil types* increased as their French proficiency increased.

In brief, the performance of both bilingual groups is predicted by their L2 experience, with L1-French L2-English listeners performing *worse* on the AL task with F0 cues as their length of stay in the US increased and with L1-English L2-French listeners performing *better* on the AL task with F0 cues as their French proficiency increased.

## Discussion and conclusion

This study first examined whether functionally monolingual French listeners and functionally monolingual English listeners differ in their ability to segment an AL that contained or did not contain F0 cues to word-final boundaries. It included both non-words and part-words in order to assess how listeners’ linguistic experience would modulate their ability to extract the probabilities of co-occurrence of two syllables (non-words) and the conditional probabilities of successive syllables (part-words) when segmenting an AL into multisyllabic units. The results showed that only monolingual French listeners benefited from F0 cues to word-final boundaries in their segmentation of the AL, to the extent that their performance in the condition with F0 cues was significantly above chance for both trials with non-word foils and trials with part-word foils. Although the monolingual French listeners’ accuracy in the condition with F0 cues could have been numerically higher, it was comparable to that observed in Tyler and Cutler (10), who also tested French listeners’ use of F0 cues to word-final boundaries, and it was comparable to the accuracy obtained in other similar studies testing the use of prosodic cues to word boundaries [5, 7]. These results thus provide another piece of evidence that speech segmentation is attuned to the role of F0 cues in the L1, in line with the findings of previous AL studies on the use of F0 cues to word boundaries [1, 4–8, 10, 11], and they further validate the use of the present experimental design with bilingual French and English listeners (for similar results, see [10]).

Importantly, this study also investigated whether listeners’ experience with an L2 learned later in life would affect their use of F0 as a cue to word boundaries in the segmentation of an AL. To do so, it compared the performance of functionally monolingual French listeners, functionally monolingual English listeners, bilingual L1-English L2-French listeners, and bilingual L1-French L2-English listeners in the use of F0 cues to word-final boundaries in an AL. The results showed that after being exposed to the AL where F0 cues signaled word-final boundaries consistent with F0 cues in French, L1-French L2-English listeners performed above chance on both foil types, whereas L1-English L2-French listeners performed above chance only on non-word foils. This pattern of results was corroborated by a three-way interaction between foil type, L1, and bilingual status, with L1-French L2-English listeners performing more poorly than monolingual French listeners on both foil types but with L1-English L2-French listeners performing better than monolingual English listeners only on non-word foils.

First and foremost, these results suggest that listeners’ experience with an L2 where F0 cues signal a different boundary from that signaled in the L1 affects their use of these cues in AL segmentation: L1-French L2-English listeners were less successful than functionally monolingual French listeners at extracting both the probabilities of co-occurrence of two syllables and the conditional probabilities of successive syllables, and L1-English L2-French listeners were more successful than monolingual English listeners at extracting the probabilities of co-occurrence of two syllables. These findings indicate that the learning of speech segmentation appears to be sufficiently *adaptive* to allow bilingual listeners to develop new routines for segmenting the L2 that they can in turn apply to the AL: For L1-French L2-English listeners, the learning of a new routine to segment English created some interference in the use of their L1 routine, which would have been more efficient to segment the AL with F0 cues; for L1-English L2-French listeners, the learning of a new routine to segment French enhanced their ability to locate probable sequences of two syllables in the AL. Additionally, the L1-French L2-English listeners’ results suggest that bilingual listeners’ use of segmentation strategies is *not selective*; instead, it shows some compromise in the degree with which either segmentation strategy is used when the two strategies conflict across the two languages. These findings indicate that bilingual listeners’ segmentation of an unfamiliar language is influenced by their experience with both the L1 and the L2, and that strategies cannot be selected as a function of how useful they are for segmenting the unfamiliar language.

The results also showed that across word types, L1-French L2-English listeners’ AL segmentation accuracy decreased as their length of stay in the US increased, and L1-English L2-French listeners’ AL segmentation accuracy increased as their French proficiency increased. These results are important, in that they provide further evidence that the observed effects of bilingual status can be attributed to bilingual listeners’ experience with the L2. Crucially, bilingual listeners’ differential performance on trials with non-word and part-word foils suggest that there are limits to the degree of influence of L2 experience on AL segmentation: In the presence of F0 cues consistent with those in French, L1-French L2-English listeners maintained their ability to extract both the probabilities of co-occurrence of two syllables (non-word foils) and the conditional probabilities of successive syllables in the presence of F0 cues (part-word foils), whereas L1-English L2-French listeners showed an ability to extract only the probabilities of co-occurrence of two syllables (non-word foils). These findings indicate that the L1 continues to have a pervasive influence on AL segmentation even in bilingual listeners, with listeners’ ability to distinguish AL words from part-word foils possibly being contingent on whether the F0 cues signal the same word boundaries in the L1.

All in all, the present study is (to our knowledge) the first to show that listeners’ experience with an L2 learned later in life affects their use of F0 as a cue to word boundaries in the segmentation of an AL, suggesting that listeners’ use of prosodic cues to word boundaries is, at least to some degree, adaptive (i.e., it is modulated by both L1 and L2 experience), and it is not selective (i.e., segmentation strategies cannot be selected as a function of how useful they are for segmenting the unfamiliar language). These findings spark interest in questions that should be investigated in further research. Among other things, it would be important to investigate bilingual listeners’ use of conflicting cues in two different ALs to determine whether poorer performance in the use of one cue (e.g., use of F0 as cue to word-final boundaries) directly translates into better performance in the use of the other cue (e.g., use of F0 as cue to word-initial boundaries).

## Supporting information

S1 File Raw data elicited from the AL segmentation task (Data.csv). The Participant column contains the participant identification code; the Group column specifies the group to which the participant belonged (EngUs = monolingual English listeners; EngFr = L1-English L2-French listeners; FrenchFrance = monolingual French listeners; FrenchUS = L1-French L2-English listeners); the L1 column contains the native language of the participant; the BilingStatus column specifies the bilingual status of the participant (no = monolingual; yes = bilingual); the Condition column specifies whether the AL contained F0 cues to word-final boundaries (NoProsody = no F0 cues to word-final boundaries; Prosody = F0 cues to word-final boundaries); the GroupProsody column is the concatenation of the Group column and the Prosody column; the Type column specifies the type of foil that the participant heard (nw = non-word foil; pw = part-word foil); the Accuracy column specifies whether the participant correctly identified the word from the AL (0 = incorrect; 1 = correct); and the Item column contains the item identification code (1 through 6 for the 6 words in the AL).
(CSV)
